# circ-Katnal1 Enhances Inflammatory Pyroptosis in Sepsis-Induced Liver Injury through the miR-31-5p/GSDMD Axis

**DOI:** 10.1155/2022/8950130

**Published:** 2022-08-08

**Authors:** Kai Kang, Nana Li, Yang Gao, Changsong Wang, Pengfei Chen, Xianglin Meng, Wei Yang, Mingyan Zhao, Kaijiang Yu

**Affiliations:** ^1^Department of Critical Care Medicine, The First Affiliated Hospital of Harbin Medical University, Harbin, 150001 Heilongjiang Province, China; ^2^Department of Critical Care Medicine, The Sixth Affiliated Hospital of Harbin Medical University, Harbin, 150028 Heilongjiang Province, China; ^3^Institute of Critical Care Medicine, The Sino Russian Medical Research Center of Harbin Medical University, Harbin, 150081 Heilongjiang Province, China

## Abstract

**Background:**

Sepsis is a systemic inflammatory response that can elicit organ dysfunction as well as circulatory diseases in serious cases. When inflammatory responses are especially dysregulated, severe complications can arise, including sepsis-induced liver injury. Various microRNAs along with circular (circ) RNAs are involved in inflammatory responses; nevertheless, their functions in regulating sepsis-induced liver injury remain unknown. The cecal ligation and puncture (CLP) procedure can induce liver injury as well as polymicrobial sepsis.

**Methods:**

In this study, CLP was used to induce liver injury as well as polymicrobial sepsis. Then, liver function, inflammatory cytokine expression, and hepatic histopathology were evaluated. High-throughput sequencing was employed to investigate the abnormal hepatic circRNA expression after CLP. Raw264.7 cells were utilized to simulation an *in vitro* sepsis inflammation model with LPS induce. The relative mRNA as well as protein levels of TNF-*α*, IL-1*β*, and IL-6 was explored by quantitative polymerase chain reaction (PCR) and enzyme-linked immunosorbent assays. We explored functional connections among circRNAs, miR-31-5p, and gasdermin D (GSDMD) using dual-luciferase reporter assays. Western blot was employed to test GSDMD, caspase-1, and NLRP3 expression in mice and cell models.

**Results:**

Our results showed that CLP-induced sepsis promoted liver injury via increasing inflammatory pyroptosis. The abnormal expression of circ-Katnal1 played an important role in CLP-induced sepsis. Downregulating circ-Katnal1 suppressed LPS-induced inflammatory pyroptosis in Raw264.7 cells. Bioinformatics and luciferase reporter results confirmed that miR-31-5p and GSDMD were downstream targets of circ-Katnal1. Inhibiting miR-31-5p or upregulating GSDMD reversed the protective effects of silencing circ-Katnal1.

**Conclusion:**

Taken together, circ-Katnal1 enhanced inflammatory pyroptosis in sepsis-induced liver injury through the miR-31-5p/GSDMD axis.

## 1. Background

Sepsis is a systemic inflammatory response syndrome elicited by polymicrobial infections; it is the primary cause for mortality among hospital patients [1]. Active, excessive, and innate immune responses are considered the primary cause of septic damage, which can lead to endothelial dysfunction, imbalanced host homeostasis, and altered metabolism due to parenchymal cellular maladjustments [2]. During sepsis, Kupffer cells activate the nuclear factor-kappa B (NF-*κ*B) signaling pathway and thereafter accelerate the production of proinflammatory cytokines such as interleukin (IL)-6, IL-1*β*, and tumor necrosis factor (TNF)-*α*, which constitute a cytokine cascade that causes tissue apoptosis and potentially multiple organ dysfunctions [3–5].

Canonical inflammasomes including NLRP3 inflammasomes activate caspase-1, while oxidized lipids and LPS trigger noncanonical inflammasomes to activate human caspase-4 and caspase-5 or mouse caspase-11 [6–8]. In the final step in inflammasome activation, the common downstream effector gasdermin D (GSDMD) is cleaved by inflammatory caspases at the junction between its N-terminal domain (GSDMD-NT) and autoinhibitory C-terminal domain (GSDMD-CT) [9–11]. GSDMD-NT binds to acidic phospholipids in the inner leaflet of the plasma membrane, which oligomerizes to create pores that disrupt plasma membrane integrity [11–13]. The proinflammatory cytokines, such as IL-18 and IL-1*β*, are released and processed by caspase-1 and induce pyroptotic cell death. However, the regulatory mechanism that controls GSDMD and influences inflammatory pyroptosis in sepsis-induced liver injury remains largely unclear.

Studies have increasingly found that circular RNAs (circRNAs) have important functions in regulating tissue microenvironments. circRNAs belong to an endogenous RNA family, have variable full-length sequences, and are identified by their covalently closed-loop structure and lack of poly-adenylated tail. circRNAs are generated by a back-splicing event, and thus are different from linear RNAs such as mRNAs. circRNAs do not have 5′ cap or 3′ tail structures [14, 15], which were discovered in the 1970s from viruses [14]. Initially, circRNAs were considered a low abundance RNA species [16]. Nevertheless, due to high-throughput sequencing and bioinformatics analyses, we now appreciate that circRNAs are substantial and common fraction of the transcriptome [16]. Previous studies have found that hsa_circRNA_104670 and hsa_circRNA_104484 might function as therapeutic targets and candidate biomarkers for sepsis [17]. circRNA VMA21 ameliorates sepsis-associated organ injury through the miR-9-3p/SMG1/inflammation axis and by regulating oxidative stress [18]. Yet, the role of circRNA1 in sepsis-associated liver injury is still largely unknown.

Thus, we examined whether circRNAs could be utilized as novel diagnostic markers for sepsis [19]. To date, there has not been an adequate study of the altered expression of circRNAs or their role in sepsis. This study investigated the circRNA expression in liver tissues that underwent sepsis-induced damage to detect their roles in GSDMD regulation during sepsis and to determine if the altered circRNA expression could lead to therapeutic targets.

## 2. Methods

### 2.1. Ethics Statement

C57BL/6 mice weighing between 15 and 20 g and aged 4 weeks (SLARC, Shanghai, China) were used in this investigation. All mice were individually housed in independent ventilated cages at 24°C to 26°C under constant humidity with a 12-h light/dark cycle. The Ethics Committee at the First Affiliated Hospital of Harbin Medical University approved all animal experiments (No. 2019-2-28).

### 2.2. *In Vivo* Sepsis Model

We induced polymicrobial sepsis in mice via cecal ligation and puncture (CLP) following the published protocols [19]. We randomly divided the 6- to 8-week-old C57BL/6 mice into three groups (6 mice in each group). Mice were anesthetized with 1.5% pentobarbital sodium solution (30 mg/kg) and underwent CLP. A 2 cm incision was made into the abdominal wall, and the cecum was exposed and ligated 0.5 cm from the tip with a 4–0 silk suture. A 22-gauge needle was used to make one puncture through the distal cecum, extruding a small amount of fecal contents. The cecum was replaced in the abdominal cavity, and the exposed abdominal wall was closed in two layers with the running 4–0 silk suture. In sham-operated mice, only laparotomy was performed, but their cecum was not ligated and punctured. The mice were resuscitated with 1 mL of normal saline subcutaneously 12 h after CLP induction. The whole blood was collected through a cardiac puncture, and the mice were sacrificed. Then, the liver tissue was collected and fixed in a 4% paraformaldehyde solution. The blood was collected from the right ventricle after thoracotomy, placed for 2 h at room temperature, and centrifuged at 1300 rpm for 20 min at 4°C. The serum was collected, and the levels of tumor necrosis factor-*α* (TNF-*α*), interleukin-6 (IL-6), interleukin-1*β* (IL-1*β*), alanine aminotransferase (ALT), and aspartate transaminase (AST) were measured.

### 2.3. High-Throughput RNA-Seq and Strand-Specific RNA-Seq Library

Total RNA from the sepsis model and sham-operated mouse liver tissues was extracted with TRIzol Reagent (Invitrogen, CA, USA). Further, ~3 *μ*g total RNA from every sample was subjected to a VAHTS Total RNA-Seq (H/M/R) Library Prep Kit (Vazyme Biotech Co., Ltd, Nanjing, China) to eliminate ribosomal RNA and retain other classes of RNAs such as noncoding RNAs and mRNAs. We treated purified RNA employing 40 U RNase R (Epicenter, New England Biolabs, MA, USA) at 37°C for 3 h, followed by TRIzol purification. Our lab used a KAPA Stranded RNA-Seq Library Prep Kit (Roche, Basel, Switzerland) to prepare an RNA-Seq library, which was subjected to deep sequencing with Illumina HiSeq 4000 (CA, USA) at Aksomics, Inc. (Shanghai, China).

### 2.4. Histological Analysis

Liver tissues were fixed with 4% paraformaldehyde solution and embedded in paraffin. Then, the sections were obtained and stained with hematoxylin and eosin (H&E) to detect hepatocyte morphology and inflammatory cell infiltration. Liver tissue samples were assessed with the transferase-mediated deoxyuridine triphosphate-biotin nick end labeling (TUNEL) staining using a fluorescence detection kit (Yeasen Biotech Co., Ltd., Shanghai, China). Five visual fields were observed in each group. The apoptotic rate in each visual field (100%) = (the total number of apoptotic cells/total cells) × 100%, and the average value were calculated.

### 2.5. Cell Culture and Transfection

We purchased RAW264.7 cells from Cell Bank in the Chinese Academy of Sciences, Shanghai, China, which were cultured in DMEM supplemented with 10% FBS in an incubator with 5% CO_2_ at 37°C. We primed RAW264.7 cells with 1 *μ*g/mL LPS for 6 h before stimulation with 5 mM ATP for 1 h. To silence circ-Katnal1, we employed control (si-NC) and circ-Katnal1-specific (si-circ-Katnal1) siRNAs, which were synthesized and designed by Shanghai Genepharma (Shanghai, China). We transfected RAW264.7 cells with si-NC and si-circ-Katnal1 utilizing Lipofectamine 3000 (Invitrogen, Carlsbad, CA, USA). After 2 d, we analyzed knockdown efficiency by western blot and real-time quantitative PCR (qPCR). For the GSDMD overexpression, the cDNA of GSDMD was cloned into the cDNA3.1 vector, which was transfected into RAW264.7 cells using Lipofectamine 3000. To overexpress or silence miR-31-5p, miR-31-5p mimic or inhibitor (Shanghai Genepharma), respectively, was transfected into RAW264.7 cells using Lipofectamine 3000.

### 2.6. Bioinformatics Analysis

A bioinformatician estimated circRNA/miRNA target genes using web-based *Circular RNA Interactome* (https://circinteractome.nia.nih.gov/). TargetScan (https://www.targetscan.org/) was used to predict the interactions between mRNAs and miRNAs.

### 2.7. miRNA and RNA Extraction and qPCR

Total RNA was extracted using TRIzol Reagent (Invitrogen) and synthesized cDNA using a pTRUEscript First Strand cDNA Synthesis Kit (Aidlab, Beijing, China). A technician performed qPCR experiments utilizing 2× SYBR Green qPCR Mix (Invitrogen) and the ABI 7900HT qPCR system (Thermo Fisher Scientific, MA, USA). Our lab detected expression changes using fold change through the 2^−*ΔΔ*CT^ method. qPCR amplifications were performed using the following primers: circ-Katnal1: forward, 5′-CCTGTCCCTGCGGAACACAG-3′ and reverse, 5′-CTTCGCATTCTCACAAATCTCC-3′; miR-31-5p: forward, 5′-CGAGGCAAGATGCTGGCA-3′ and reverse, 5′-AGTGCAGGGTCCGAGGTATT-3′; GSDMD: forward, 5′-ATATCTGCCAGAGATTGATA-3′ and reverse, 5′-TGGAAGTATCTTTGCCGGT-3′; U6: forward, 5′-ATTGGAACGATACAGAGAAGATT-3′ and reverse, 5′-GGAACGCTTCACGAATTTG-3′; and GAPDH forward 5′-GCAAGGATGCTGGCGTAATG-3′ and reverse 5′-TACGCGTAGGGGTTTGACAC-3′. We normalized circ-Katnal1 and GSDMD expression levels to GAPDH and miR-873 expression levels to U6.

### 2.8. Dual-Luciferase Reporter Assay

293 T cells were cotransfected with 150 ng empty pmiR-GLO-NC or pmiR-GLO-circ-Katnal1-wt or with pmiR-GLO-circ-Katnal1-mut, pmiR-GLO-GSDMD-wt, or pmiR-GLO-GSDMD-mut (Sangon Biotech, Shanghai, China) and with 2 ng internal control pRL-TK (Promega, WI, USA). The technician cotransfected 293 T cells with pPG-miR-miR-31-5p or pPG-miR-NC. The dual-luciferase reporter assay kit was applied to detect luciferase activity following manufacturer's protocols (Promega, Beijing, China). Relative luciferase activity was normalized to Renilla luciferase.

### 2.9. Western Blot Analysis

Lysis buffer comprising 10 mM Tris-buffer (pH 7.6), 1 mM PMSF, 1% phosphatase inhibitor cocktail, and 1% Triton X-100 was used to lyse cells. We boiled cell lysates in SDS sample buffer, which were resolved on 10% SDS-PAGE gel. After transfer to membranes, we incubated immunoblots overnight with primary antibodies against NLRP3 (Santa Cruz Biotechnology, TX, USA), GSDMD (Abcam, Cambridge, UK), GAPDH (Proteintech, IL, USA), and caspase-1 (Santa Cruz Biotechnology). Immunoreactive bands were visualized with an ECL detection reagent (Millipore, MA, USA).

### 2.10. Serum ALT and AST Measurements

Serum AST and ALT levels were detected utilizing assay kits (Nanjing Jiancheng Bioengineering Institute) following the manufacturer's protocol.

### 2.11. Enzyme-Linked Immunosorbent Assay (ELISA) for Inflammatory Factors

Blood was collected from the right ventricle after thoracotomy. After being placed for 2 h at room temperature, the blood was centrifuged at 1300 rpm for 20 min at 4°C, and the serum was collected. The levels of IL-1*β*, IL-6, or TNF-*α* in the serum were measured using inflammatory factor ELISA kits (eBioscience, San Diego, CA, USA) following the protocols. The results were determined spectrophotometrically using a microplate reader.

### 2.12. Statistical Analysis

Data were shown as means ± standard deviation (SD). Statistical analyses were performed in GraphPad Prism 5.0 (GraphPad Software Inc., La Jolla, CA, USA) to determine significant differences among groups. *P* values ≤0.05 indicated statistically significant differences. Two-tailed Student *t*-tests were used to determine significant differences between the two groups, and one-way ANOVA with post hoc Bonferroni tests was used to determine significant differences among three or more groups.

## 3. Results

### 3.1. CLP-Induced Sepsis Promoted Liver Injury through Increased Inflammatory Pyroptosis

**Figure 1:**
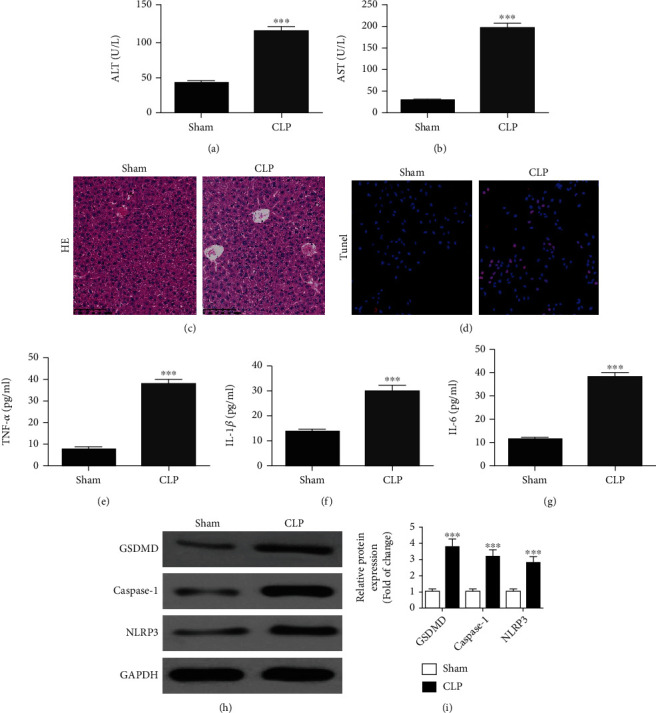
CLP-induced sepsis promoted liver injury by increasing inflammatory pyroptosis. Mice were sacrificed 12 h after CLP, and liver tissues and serum were collected for further analysis. (a, b) ALT and AST expression levels were measured using ELISA. Data were presented as mean ± SD. ^∗∗∗^*P* < 0.001, versus the sham group. (c, d) HE and TUNEL staining showed the liver injury (magnification, ×200). (e)–(g) ELISA detection showed the expression of inflammatory factors TNF-*α*, IL-1*β*, and IL-6. Data were presented as mean ± SD. ^∗∗∗^*P* < 0.001, versus the sham group. (h, i) Western blot detection showed GSDMD, caspase-1, and NLRP3 expression. Data were presented as mean ± SD. ^∗∗∗^*P* < 0.001, versus the sham group.

To examine liver injury resulting from sepsis, we investigated two biochemical serum markers of liver function, ALT and AST. Following CLP-induced sepsis, serum ALT and AST levels were elevated (Figures 1(a) and 1(b)). H&E (Figure 1(c)) staining showed that the liver cells from the sham group exhibited an intact cellular structure with round, large, and clearly visible nuclei. In contrast, CLP-induced sepsis showed obvious cell damage with irregularly shaped cells, concentrated cytoplasm, and nuclei. TUNEL (Figure 1(d)) staining showed that CLP-induced sepsis promoted liver injury and cell death. ELISA showed that CLP-induced sepsis promoted the expression of inflammatory factors TNF-*α*, IL-1*β*, and IL-6 (Figures 1(e) and 1(g)). The western blot showed that CLP-induced sepsis promoted GSDMD, caspase-1, and NLRP3 expression (Figures 1(h) and 1(i)). These data demonstrated that CLP-induced sepsis promoted liver injury by inducing liver pyroptosis.

### 3.2. Abnormal circ-Katnal1 Expression Was Important for CLP-Induced Sepsis

**Figure 2:**
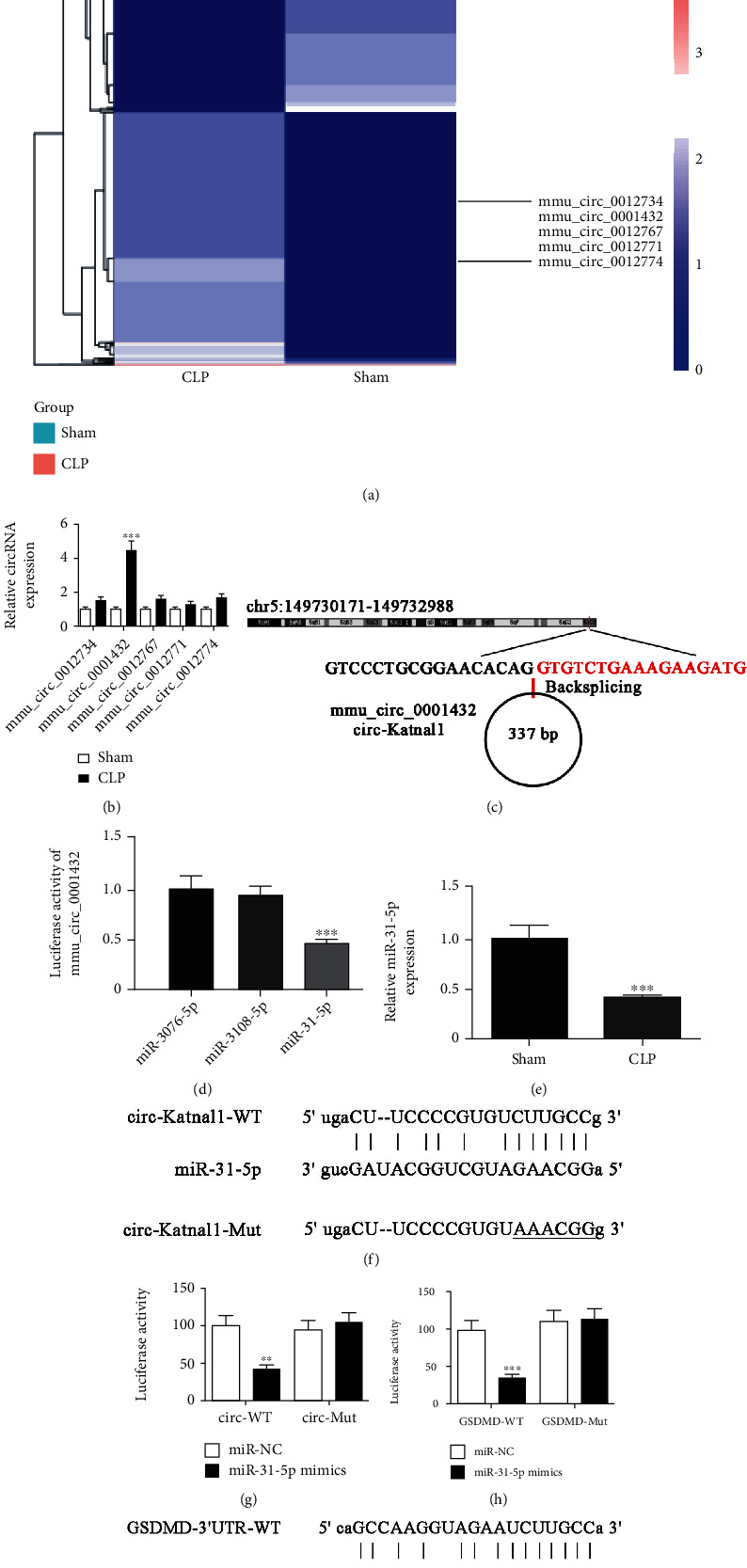
Abnormal expression of circ-Katnal1 played an important role in CLP-induced sepsis. (a) Heat map showing the expression of circRNA following 12-h induction after CLP in liver tissues. (b) RT-qPCR detection showed the expression of mmu_circ_0012734, mmu_circ_0001432, mmu_circ_0012767, mmu_circ_0012771, and mmu_circ_0012774 in normal and CLP-induced mouse liver tissues. Data were presented as mean ± SD. ^∗∗∗^*P* < 0.001, versus the sham group. (c) Genomic loci of the *Katnal1* gene and mmu_circ_0001432 (circ-Katnal1). (d) The luciferase activity of circ-Katnal1 in HEK293 T cells transfected with different miRNA mimics, which were putative binding sites for the circ-Katnal1 sequence. Luciferase activity was normalized by Renilla luciferase activity. Data were presented as mean ± SD. ^∗∗∗^*P* < 0.001. (e) RT-qPCR detection showed the expression of miR-31-5p in normal and CLP-induced mouse liver tissues. Data were presented as mean ± SD. ^∗∗^*P* < 0.01 versus the sham group. (f, g) Prediction of the binding sites of miR-31-5p in circ-Katnal1. The MUT version of circ-Katnal1 is presented. The relative luciferase activity determined 48 h after the transfection of HEK293 T cells with miR-31-5p mimic/NC or circ-Katnal1 WT/Mut. Data were presented as means ± SD. ^∗∗^*P* < 0.01. (h, i) Prediction of the binding sites of miR-31-5p in 3′UTR-GSDMD. The MUT version of 3′UTR-GSDMD is presented. Relative luciferase activity determined 48 h after the transfection of HEK293 T cells with miR-31-5p mimic/NC or 3′UTR-GSDMD WT/Mut. Data were presented as means ± SD. ^∗∗∗^*P* < 0.001.

An increasing number of studies found that ncRNAs had indispensable functions in regulating tissue microenvironments [20, 21]. Studies have also suggested that circRNAs might function to regulate patient immune systems against various pathogens such as viruses and bacteria. Mounting evidence has revealed that circRNA dysregulation is an early event in various conditions such as sepsis [22]. We employed high-throughput RNA-Seq to identify abnormally expressed circRNAs between liver tissues of septic and sham-operation mice. The results showed that CLP-induced sepsis resulted in abnormal circRNA expression in liver tissues, including increased expression of mmu_circ_0012734, mmu_circ_0001432, mmu_circ_0012767, mmu_circ_0012771, and mmu_circ_0012774 (Figure 2(a)). qPCR data confirmed that the mmu_circ_0001432 expression was significantly increased in liver tissues of CLP-induced septic mice compared with sham-operation mice (Figure 2(b)). This finding suggested that mmu_circ_0001432 played an important role in CLP-induced sepsis.

Bioinformatics analysis (http://www.circbase.org/) revealed that mmu_circ_0001432 was cyclized and derived from an exon of the *Katnal1* gene, which is located at chr5:149730171-149732988. Thus, mmu_circ_0001432 was also named circ-Katnal1 (Figure 2(c)).

Analysis of starBase suggested that mmu_circ_0001432 could interact with miR-3076-5p, miR-3108-5p, and miR-31-5p. Luciferase reporter assays showed that only miR-31-5p could inhibit luciferase activity from the circ-Katnal1 plasmid (Figure 2(d)), suggesting that miR-31-5p was the circ-Katnal1 downstream target. qPCR data showed that the miR-31-5p expression decreased in liver tissue from the CLP-induced sepsis mouse model (Figure 2(e)). Luciferase reporter results validated that miR-31-5p suppressed luciferase activity of the pmiR-GLO-circ-Katnal1-wt plasmid but not of the pmiR-GLO-circ-Katnal1-mut plasmid (Figures 2(f) and 2(g)). The results suggested that miR-31-5p was the circ-Katnal1 target.

Bioinformatics analyses revealed that GSDMD was the miR-31-5p downstream target. To verify the correlations between GSDMD and miR-31-5p, mutated or wild-type 3′UTR-GSDMD sequences, including the miR-31-5p binding sequence, were constructed and inserted into the luciferase reporter vector (pmiR-GLO-GSDMD-wt and pmiR-GLO-GSDMD-mut, respectively, Figure 2(i)). After transfecting these luciferase reporter vectors into 293 T cells with/out miR-31-5p mimic, the luciferase reporter results showed that miR-138-5p suppressed luciferase activity from the wild-type but not the mutated reporter (Figure 2(h)). Thus, these data demonstrated that miR-31-5p is a GSDMD target.

### 3.3. Silencing circ-Katnal1 Suppressed LPS-Induced Inflammatory Pyroptosis in RAW264.7 Cells

**Figure 3:**
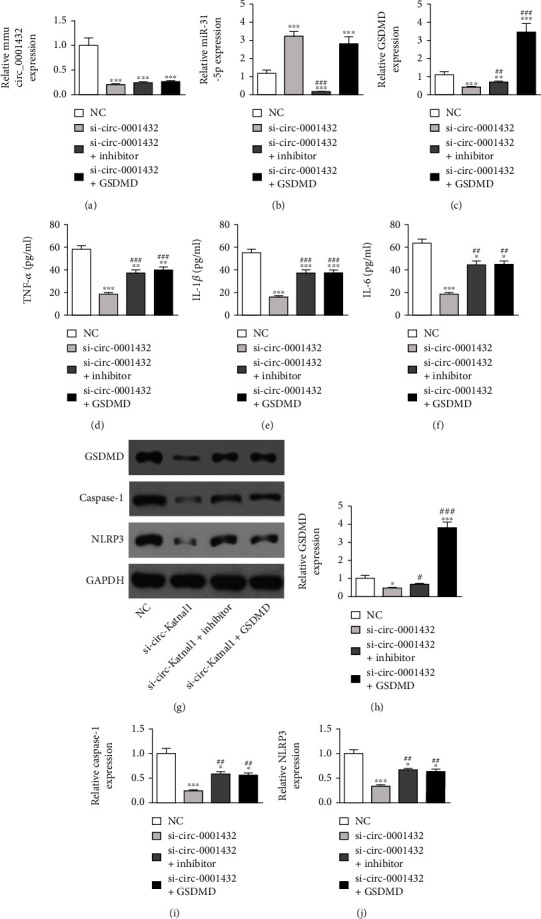
Downregulation circ-Katnal1 suppressed LPS-induced inflammatory pyroptosis in RAW264.7 cells. However inhibiting miR-31-5p or upregulating GSDMD reversed the protective effect after circ-Katnal1 silencing. (a)–(c) RT-qPCR detection showed the expression of circ-Katnal1, miR-31-5p, and GSDMD. (d)–(f) ELISA detection showed the expression of inflammatory factors TNF-*α*, IL-1*β*, and IL-6 under LPS condition (1 *μ*g/mL). Data were presented as mean ± SD. ^∗^*P* < 0.05, ^∗∗^*P* < 0.01, ^∗∗∗^*P* < 0.001, versus sham NC. ^##^*P* < 0.01, ^###^*P* < 0.001, versus si-circ-Katnal1 (si-mmu_circ_0001432). (g)–(j) Western blot detection showed GSDMD, caspase-1, and NLRP3 expression. Data were presented as mean ± SD. ^∗∗∗^*P* < 0.001, versus the sham group. Data were presented as mean ± SD. ^∗^*P* < 0.05, ^∗∗∗^*P* < 0.001, versus sham NC; ^#^*P* < 0.05, ^##^*P* < 0.01, ^###^*P* < 0.001, versus si-circ-Katnal1 (si-mmu_circ_0001432).

RT-qPCR data illustrated that the circ-Katnal1 expression was decremented after transfecting the siRNA against circ-Katnal1 (si-circ-Katnal1). However, neither treatment with miR-31-5p inhibitor nor increasing the GSDMD expression altered the circ-Katnal1 expression (Figure 3(a)), suggesting that miR-31-5p and GSDMD were downstream of circ-Katnal1. qPCR results then revealed that silencing circ-Katnal1 increased the miR-31-5p expression. Overexpressing GSDMD had no effect on the increased miR-31-5p expression following si-circ-Katnal1 treatment (Figure 3(b)). These data suggested that miR-31-5p was circ-Katnal1 downstream and independent of GSDMD. The result shows that silencing circ-Katnal1 decremented the GSDMD expression. However, downregulating miR-31-5p reversed the inhibitory effect of si-circ-Katnal1 upon the GSDMD expression. After transfecting the GSDMD overexpression vector, GSDMD expression increased significantly (Figure 3(c)). In summary, the data suggested that silencing circ-Katnal1 decreased GSDMD levels by promoting the miR-31-5p expression.

The detection of inflammatory factors showed that LPS pretreatment promoted IL-1*β*, TNF-*α*, and IL-6 secretion. However, inhibiting miR-31-5p or overexpressing GSDMD restored the IL-6, IL-1*β*, and TNF-*α* secretion to below levels of LPS stimulation (Figures 3(d)–3(f)). Western blot showed that inhibiting miR-31-5p or overexpressing GSDMD restored the expression of GSDMD, caspase-1, and NLRP3 to levels below those of LPS stimulation (Figures 3(g)–3(j)). Together, these findings suggest that downregulating circ-Katnal1 could suppress LPS-induced inflammatory pyroptosis in RAW264.7 cells.

### 3.4. Overexpressing GSDMD Reversed Protective Effects of miR-31-5p on LPS-Induced Inflammatory Pyroptosis in RAW264.7 Cells

**Figure 4:**
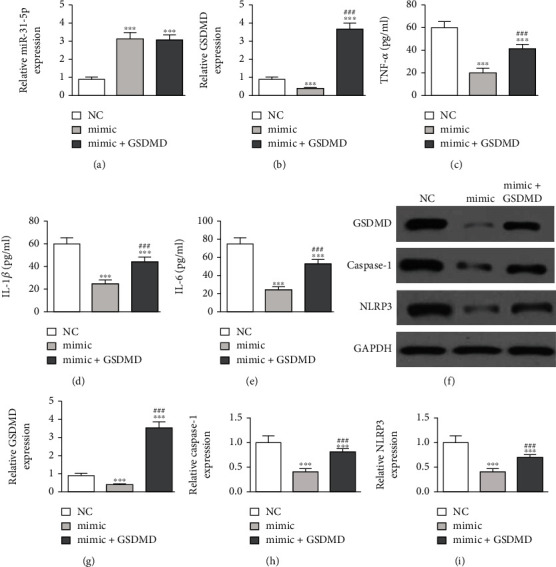
Upregulation of GSDMD reversed the protective effect of miR-31-5p on LPS-induced inflammatory pyroptosis in RAW264.7 cells. (a, b) RT-qPCR detection showed the expression of miR-31-5p and GSDMD. (c)–(e) ELISA detection showed the expression of inflammatory factors TNF-*α*, IL-1*β*, and IL-6 under LPS pretreatment (1 *μ*g/mL). Data were presented as mean ± SD. ^∗∗∗^*P* < 0.001, versus sham NC. ^###^*P* < 0.001, versus mimic. (f)–(i) Western blot detection showed the expression of GSDMD, caspase-1, and NLRP3 expression. Data were presented as mean ± SD. ^∗∗∗^*P* < 0.001, versus sham NC. ^###^*P* < 0.001, versus mimic.

Our qPCR data showed that the miR-31-5p expression incremented after transfecting the miR-31-5p mimic. Overexpressing GSDMD did not affect miR-31-5p levels (Figure 4(a)), suggesting that GSDMD was downstream of miR-31-5p. qPCR data also showed that overexpressing miR-31-5p decremented the miR-31-5p expression. Finally, after transfecting the GSDMD overexpression vector, the GSDMD expression was significantly increased (Figure 4(b)). Together, the results suggested that miR-31-5p was a negative regulator for the GSDMD expression.

Measuring levels of inflammatory factors showed that LPS pretreatment promoted TNF-*α*, IL-6, and IL-1*β* secretion. Overexpressing miR-31-5p decremented the expression of these inflammatory factors. Overexpressing GSDMD restored TNF-*α*, IL-1*β*, and IL-6 to levels below those of LPS treatment (Figures 4(c)–4(e)). Western blot data showcased that overexpressing GSDMD restored GSDMD, caspase-1, and NLRP3 expression after LPS pretreatment and miR-31-5p overexpression (Figures 4(f)–4(i)). Thus, overexpressing GSDMD reversed the protective effects of miR-31-5p on LPS-induced inflammatory pyroptosis in RAW264.7 cells.

## 4. Discussion

The liver is a major target during sepsis. After exposure to microbial pathogens, the liver displays sepsis-induced injury, inflammation, and cell death [23]. From histological analysis, TUNEL staining, and serum ALT and AST measurements, CLP-induced sepsis increased liver injury. CLP-induced sepsis can produce inflammatory mediators including IL-6, TNF-*α*, and IL-1*β*, which aggravate inflammation [24, 25]. In the CLP-induced sepsis model, these factors aggravated liver cell death and inflammation.

Previous research has revealed that hepatic pyroptosis correlates with CLP-induced liver injury [26]. Pyroptosis mediated by caspase-1, GSDMD, and inflammatory signals activates immune responses in the liver [27]. GSDMD cleavage by inflammatory caspases elicits pyroptotic cell death [10]. GSDMD is a substrate of caspases whose cleavage is at the predicted site during inflammasome activation. It is demanded for pyroptosis and IL-1*β* secretion. The GSDMD-NT expression triggers inflammatory death [11, 28]. Suppressing pyroptosis protects mice from CLP-induced death [29]. It has also been discovered that CLP stimulates liver pyroptosis by inducing the abnormal expression of circRNAs in the tissue microenvironment.

We discovered that mmu_circ_0001432 (circ-Katnal1) was abnormally expressed in a murine CLP-induced sepsis model. Downregulating circ-Katnal1 suppressed LPS-induced inflammatory pyroptosis in RAW264.7 cells. Macrophages circulating in blood or residing in tissues represent the first barrier against external infection through controlling both innate and acquired immunity. Because of their diversity and plasticity, macrophages undergo heterogeneous activation and polarization. M1 macrophages release proinflammatory molecules, which cause tissue damage [30]. Luciferase reporter assays showed that miR-31-5p was a downstream target of circ-Katnal1. Previous studies showed a decrease in the miR-31-5p expression in mice with sepsis [31]. The upregulation of miR-31-5p inhibited inflammatory cytokine expression [32, 33]. In this study, we also found that the miR-31-5p expression decreased in mice with CLP. The downregulation of circ-Katnal1 promoted the miR-31-5p expression. However, inhibiting miR-31-5p reversed the protective effects of silencing circ-Katnal1 on LPS treatment in RAW264.7 cells.

The luciferase reporter assays showed that GSDMD was the downstream target of miR-31-5p. The downregulation of circ-Katnal1 decreased the GSDMD expression. The upregulation of GSDMD reversed the protective effects of silencing circ-Katnal1 on LPS treatment in RAW264.7 cells. Previous studies found that the activation of proinflammatory caspases and their subsequent cleavage of gasdermin D (GSDMD) led to inflammatory apoptosis [34, 35]. Upregulating GSDMD reversed the protective effects of miR-31-5p on LPS-induced inflammatory pyroptosis in RAW264.7 cells. These findings suggested that circ-Katnal1 enhanced inflammatory pyroptosis in sepsis-induced liver injury through the miR-31-5p/GSDMD axis.

## 5. Conclusions

In summary, downregulating circ-Katnal1 attenuated proinflammatory cytokine production and inflammatory pyroptosis during CLP-induced sepsis via upregulating miR-31-5p and downregulating GSDMD. This study indicated that circ-Katnal1 was a promising therapeutic biomarker for sepsis-induced liver injury.

## Data Availability

The datasets used and/or analyzed during the current study are available from the corresponding authors on reasonable request.
